# Serum Golgi protein 73 is a marker comparable to APRI for diagnosing significant fibrosis in children with liver disease

**DOI:** 10.1038/s41598-018-34714-y

**Published:** 2018-11-13

**Authors:** Langli Liu, Jianwen Wang, Jiayan Feng, Mingjie Yao, Chenzhi Hao, Yijie You, Yanyan Yan, Jingyu Gong, Yi Lu, Xinbao Xie, Meihong Zhang, Lian Chen, Tingting Li, Fengmin Lu, Jian-She Wang

**Affiliations:** 1 The Department of Pediatrics, Jinshan Hospital, Fudan University, Shanghai, 201508 China; 2 Department of Microbiology & Infectious Disease Center, School of Basic Medicine, Peking University Health Science Center, Beijing, 100191 China; 3 The Department of Pathology, Children’s Hospital of Fudan University, Shanghai, 201102 China; 4 The Center for Pediatric Liver Diseases, Children’s Hospital of Fudan University; Department of Pediatrics, Shanghai Medical College of Fudan University, Shanghai, 201102 China; 5 Department of Biomedical Informatics, School of Basic Medical Sciences, Peking University Health Science Center, Beijing, 100191 China

## Abstract

Serum Golgi protein 73 (GP73) is a promising marker for significant fibrosis in adults. However, current diagnostic value of serum GP73 for liver fibrosis in children is unknown. To investigate the relationship between levels of serum GP73 and liver fibrosis in children, we measured serum GP73 in 86 healthy controls and 183 patients with liver diseases using commercially available double-antibody sandwich enzyme-linked immunosorbent assay (ELISA) kit. The value of serum GP73 in fibrosis stage assessment was compared with aspartate transaminase to platelet ratio index (APRI). We found that serum GP73 was decreasing with age in healthy controls, while it was increasing with the extent of inflammation and fibrosis in patients with liver diseases. Though area under the receiver operating curve (AUROC) of serum GP73 for diagnosing significant fibrosis was nearly equal to APRI (0.62 vs 0.64) in patients aged 3 years or older, AUROC for serum GP73 was superior to APRI (0.76 vs 0.67) in patients aged below 3 years, indicating that serum GP73 is comparable to APRI for diagnosing significant fibrosis in children.

## Introduction

Chronic liver diseases, including genetic diseases, viral hepatitis, autoimmune hepatitis, and congenital malformations can lead to liver fibrosis and eventually to cirrhosis in children. In the USA, it is estimated that each year approximately 15,000 children are hospitalized for liver diseases and most of them may have to receive liver transplantation^1^. Furthermore, several pediatric liver diseases may continue into adulthood and lead to cirrhosis or hepatocellular carcinoma. Recent studies have demonstrated that liver fibrosis can be reversible when the underlying cause of liver injury is removed^2–5^. Therefore, precise assessment of liver fibrosis is crucial for prognosis and long-term monitoring in children. Liver biopsy has long been considered a gold diagnostic standard for assessing liver fibrosis, but it is difficult to implement in children because of its limitations such as invasiveness, non-repeatability, complication, and sampling errors^6,7^. Consequently, alternative methods which are non-invasive, repeatable, and cheaper for evaluating liver disease progression have always been pursued by clinicians.

In recent years, serum GP73 has been widely used for diagnosing hepatocelluar carcinoma (HCC) and monitoring progression of liver diseases in adults^8–12^. However, some researchers believed that serum GP73 was unlikely to be very specific for diagnosing hepatocellular cancer but it was probably a promising surrogate for liver fibrosis^13^. Several studies showed that GP73 might be a potential marker for diagnosing significant fibrosis and cirrhosis in adults^14–16^. Since there is no difference in diagnosing liver fibrosis between adults and children, and so far, no report in children on the relationship between serum GP73 levels and the progression of liver fibrosis was seen. The present study was designed to explore the value of serum GP73 levels in diagnosing significant fibrosis in children.

## Results

### Serum levels of GP73 decreased with age

**Figure 1:**
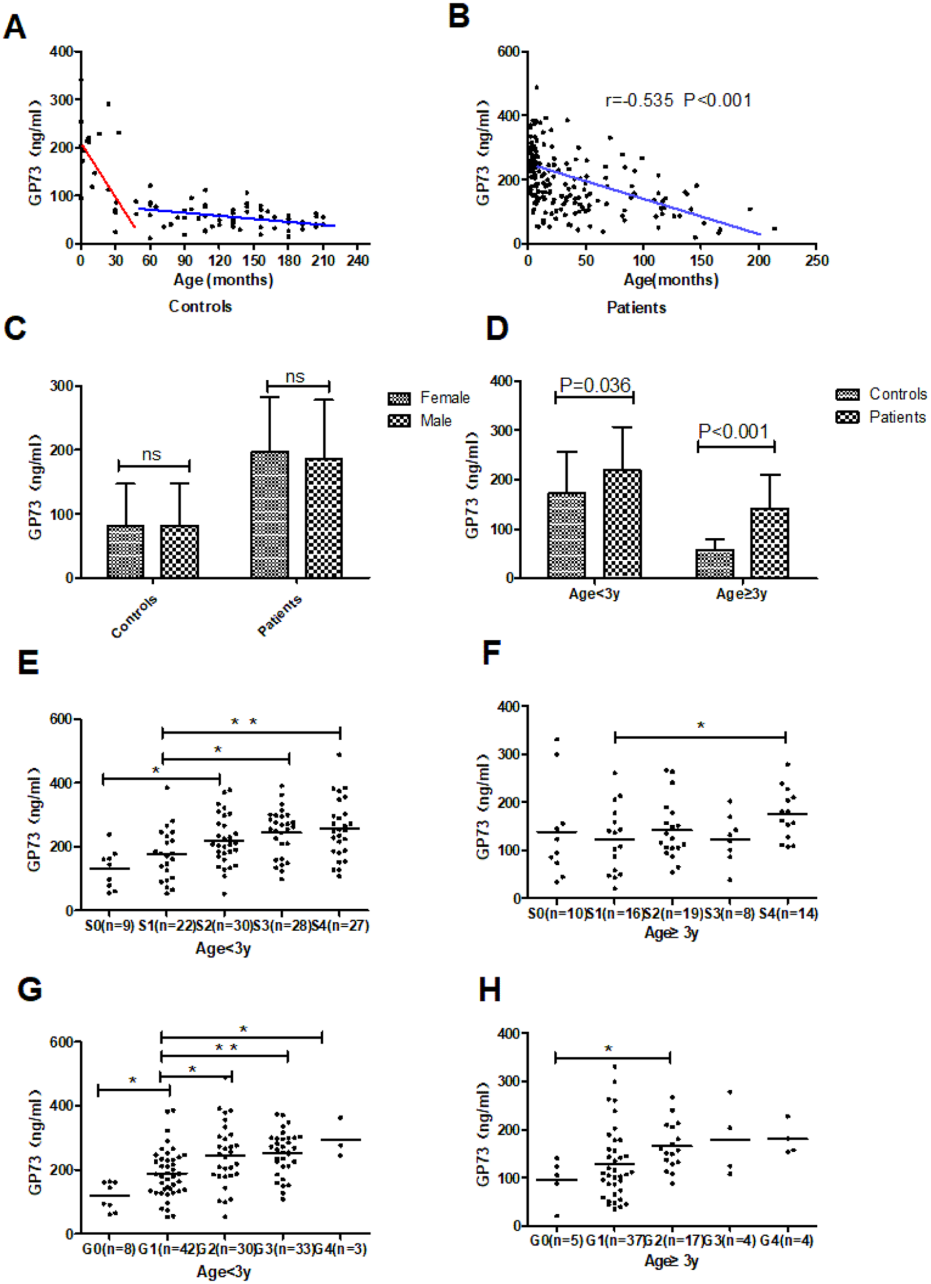
The correlation between serum GP73 and ages, gender, hepatic fibrosis stages, hepatic inflammatory grades. The correlation between serum GP73 and ages in controls (**A**) and patients (**B**), respectively. (**C**) The relationship between serum GP73 and gender in controls and patients, respectively. (**D**) The comparison of serum GP73 between patients and children in aged below 3 years and 3 years or older. The relationship of serum GP73 with hepatic fibrosis stages in patients aged below 3 years (**E**) and 3 years or older (**F**), respectively. The relationship of serum GP73 with hepatic inflammatory grades in patients aged below 3 years (**G**) and 3 years or older (H), respectively. ns = no significance, *P < 0.05, **P < 0.001.

Figure 1A showed the serum levels of GP73 exhibited a decreasing trend with age in healthy controls. We also noticed that the level of serum GP73 in subgroup aged less than three years was significantly higher than that of in subgroup aged three years or older (172.9 ± 84 vs 56.3 ± 23.4, P < 0.001). Correlation analysis revealed that the level of serum GP73 was negatively correlated with ages in both healthy subgroups: aged less than 3 years (r = −0.479, P = 0.038), and aged three years or older (r = −0.313, P = 0.001). The same trend was observed in patients (r = −0.535, P < 0.001, Fig. 1B). No significant differences of serum GP73 levels were observed between genders either in controls or patients (Fig. 1C).

To eliminate the influence of age, patients was further divided into two subgroups including aged less than 3 years, and three years or older.

### Serum levels of GP73 were positively associated with liver fibrosis stage and hepatic inflammation grade in patients

There were significant differences of GP73 levels between patients with liver disease and healthy controls (Fig. 1D). Compared with healthy controls, serum levels of GP73 significantly increased in subgroup aged below 3 years (218.7 ± 88.0 vs 172.9 ± 84.2, P = 0.036), and subgroup aged three years or older (141.4 ± 68.8 vs 56.3 ± 23.4, P < 0.001).

To explore if serum GP73 level was a reflection of the severity of fibrosis, partial correlation analysis between serum levels of GP73 and fibrosis stage were performed. When adjusted for age, serum GP73 was positively correlated with hepatic fibrosis stages (r = 0.338, P < 0.001). Because inflammation may always accompany of fibrosis, its correlation with serum GP73 was also measured. And results showed that serum GP73 was also positively correlated with hepatic inflammatory grades (r = 0.412, P < 0.001). But it should be noticed that the extent of correlation between serum GP73 and fibrosis or inflammation is different in two age subgroups. Simple scatter plot showed that serum levels of GP73 were gradually elevated with progression of liver fibrosis and this was more obviously in subgroup aged less than 3 years (Fig. 1E,F). No statistically significance was observed in subgroup aged 3 years or older. Patients were divided into significant fibrosis (S2-S4) and no/minor fibrosis (S0-S1). Serum levels of GP73 was significantly higher in patients with significant fibrosis compared to patients with no/minor fibrosis in the subgroup aged less than 3 years (239 ± 82.3 ng/ml vs 162 ± 78.5 ng/ml, P < 0.001) (Table 1). No significant difference between no/minor fibrosis and significant fibrosis in the subgroup aged 3 years or older was observed (128.4 ± 81.7 ng/ml vs 149.6 ± 58.8 ng/ml, P = 0.22, Table 1).

**Table 1:** The comparison of clinic and laboratory characteristic in patients between hepatic significant fibrosis and no/minor fibrosis.

Variables	Age <3years (n = 116)			Age ≥ 3years (n = 67)		
	S0-1(n = 31)	S2-4(n = 85)	P-Value	S0-1(n = 26)	S2-4(n = 41)	P-Value
Age (month)	17.1 ± 10.8	10 ± 8.5	P < 0.001	89.4 ± 51.7	81.9 ± 35.7	P = 0.48
GP73 (ng/ml)	162 ± 78.5	239 ± 82.3	P < 0.001	128.4 ± 81.7	149.6 ± 58.8	P = 0.22
TB (umol/L)	19.1 ± 30.6	142 ± 108	P < 0.001	48 ± 65	43.3 ± 70.8	P = 0.61
ALT (U/L)	207 ± 184	257 ± 296	P = 0.493	203 ± 460	162 ± 182	P = 0.62
AST (U/L)	169 ± 157	355 ± 347	P = 0.002	117.6 ± 136	176.5 ± 192	P = 0.12
ALB (g/L)	43.3 ± 2.3	40.2 ± 4.7	P = 0.001	44.3 ± 3.1	41.7 ± 5.2	P = 0.028
GGT (U/L)	93.8 ± 114	140 ± 179	P = 0.016	209.6 ± 378	109.7 ± 144	P = 0.425
TBA (umol/l)	42 ± 106	180 ± 111	P < 0.001	98.6 ± 164	69.8 ± 111.5	P = 0.62
PLT (10*9/L)	299 ± 146	326 ± 166	P = 0.419	225.8 ± 95	223 ± 112	P = 0.918
Inflammation	0.94 ± 0.73	2.2 ± 0.88	P < 0.001	1 ± 0.6	1.8 ± 1	P = 0.001

Abbreviation: GP73 = Golgi protein 73, TB = Total bilirubin, ALT = Alanine transaminase, AST = Aspartate transaminase, ALB = Serum albumin, GGT = Gamma glutamyl transpeptidase, TBA = Total bile acid, PLT = Platelet. P values were calculated by student’s t-test and Mann-Whitney U test.

For the relationship of serum GP73 and hepatic inflammatory grades, a noticeable increase of serum GP73 level in parallel with the increase of hepatic inflammatory grades was observed in both age groups (Fig. 1G,H). Similar to fibrosis stages, patients were divided into significant inflammation (G2-G4) and no/minor inflammation (G0-G1). Serum GP73 level was significantly higher in patients with significant inflammation than that of patients with no/minor inflammation in subgroup aged less than 3 years (250.5 ± 82.9 vs 176.9 ± 76.9, P < 0.001) and subgroup aged 3 years or older (169.7 ± 50.6 vs 124.5 ± 70.1, P < 0.01).

### Sensitivity and specificity of serum GP73 for diagnosing significant fibrosis

**Figure 2:**
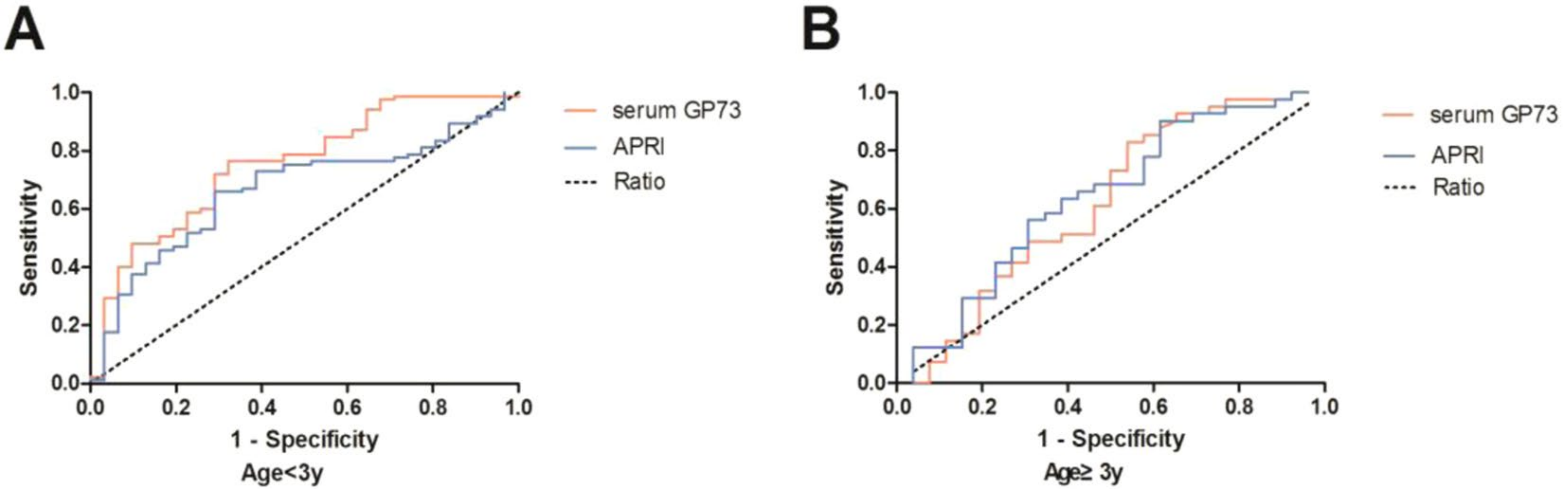
ROC curves of serum GP73 and APRI for diagnosing significant fibrosis. (**A**) The area under the ROC curve (AUC) for GP73 is 0.76 (95% CI 0.66 to 0.86), for APRI is 0.67 (95% CI 0.56 to 0.77) in patients aged less than 3 years. (**B**) The AUC for GP73 is 0.62 (95% CI 0.47 to 0.77), for APRI is 0.64 (95% CI 0.5 to 0.78) in patients aged 3 years or older.

To determine the diagnostic value of serum GP73 for significant fibrosis in children with liver disease, the ROC curve was plotted in patients aged below 3 years and aged 3 years or older, respectively. With a cut-off value set at 179.6 ng/ml, the area under the ROC curve (AUC) for serum GP73 was 0.76 (95% CI: 0.66–0.86), comparing to the AUC 0.67 (95%CI: 0.56–0.77) for APRI in patients aged less than 3 years. The sensitivity and specificity of serum GP73 were 76.5% and 67.7%, respectively (Fig. 2A). With a cut-off value set at 102.9 ng/ml in patients aged 3 years or older, the AUC for serum GP73 was 0.62 (95%CI: 0.47–0.77) with a sensitivity of 82.9% and a specificity of 46.2%; while the AUC for APRI was 0.64 (95%CI: 0.5–0.78) with the sensitivity of 90.2% and specificity of 38.5% (Fig. 2B).

### Association between serum levels of GP73 and liver function indices

To investigate if serum GP73 levels reflect the degree of liver damage, we compared serum GP73 with common liver function markers, such as ALT, AST, TBA, and TB. Partial correlation analysis showed that serum GP73 was positively correlated with TB (r = 0.455, P < 0.001), ALT (r = 0.206, P < 0.007), AST (r = 0.354, P < 0.001), and TBA (r = 0.413, P < 0.001), while negatively correlated with ALB (r = −0.465, P < 0.001) (Table 2). Additionally, we compared patients with no/minor fibrosis to advanced fibrosis, and analyzed the correlation between GP73 and other common liver function parameters. Serum GP73 was more significantly correlated with TB (r = 0.425, P < 0.001), and ALB (r = −0.43, P < 0.001) in patients with advanced fibrosis than in patients with no or minor fibrosis (r = 0.174, P = 0.200 for TB; r = −0.256, P = 0.057 for ALB). However, the levels of serum GP73 was more closely correlated with ALT (r = 0.39, P < 0.01) and AST (r = 0.577, P < 0.001) in patients with no or minor fibrosis than in patients with advanced fibrosis (r = 0.163, P = 0.069 for ALT; r = 0.263, P = 0.003 for AST).

**Table 2:** Partial correlation analysis between serum GP73 and liver function indices.

Variables	Serum GP73 (ng/ml)	
	R	P-Value
TB (umol/L)	0.445	<0.001
ALT (U/L)	0.237	0.001
AST (U/L)	0.356	<0.001
ALB (g/L)	−0.427	<0.001
GGT (U/L)	0.055	0.465
TBA (umol/l)	0.419	<0.001
PLT (10^9/L)	−0.083	0.265

## Discussion

Golgi protein 73 (GP73), a type II transmembrane protein, was first discovered in patient with adult giant-cell hepatitis^17^. Its expression is significantly increased in advanced liver diseases, especially in hepatic cirrhosis^15,18,19^. Though the function of GP73 remains unclear, some researchers believed GP73 expression represented the degree of hepatic fibrosis, for its expression in activated stellate cells^18^, the main factor and trigger for hepatic fibrosis. Other liver-specific biomarkers for fibrosis, such as hyaluronic acid (HA), laminin (LN), and type III procollagen protein (PCIII) were also reported to be positively correlated with GP73, indicating its potential role for diagnosing liver fibrosis^12^. Regardless of etiology, GP73 expression was dramatically upregulated in chronic liver diseases^20,21^. In addition, up-regulation of GP73 expression was reversible during regression of fibrosis^18,19,22^, implying serum GP73 may be a promising marker for monitoring progression of liver diseases. However, no studies related to GP73 in childhood liver disease were found.

In our present study, we interestingly found that serum GP73 was strikingly affected by age. It decreased in older individuals, no matter in healthy controls, or in patients with chronic liver disease. It is uncertain which mechanism lead to alteration of serum GP73 with age. Previous study showed that increased expression of serum GP73 was detected in various liver diseases^18,20,23^, and hepatocytes are derived from epithelial progenitor cell during normal embryological development. So, we speculated that the high levels of serum GP73 in infant and young children may be associated with more active hepatocyte differentiation or proliferation. Our finding that GP73 differentially expressed with age made it difficult as an ideal diagnostic parameter.

However, when dividing patients into aged 3 years or older and aged less than 3 years, the relationship between serum GP73 with fibrosis and inflammation turned out to be different. We demonstrated that serum levels of GP73 elevated gradually with increased staging of hepatic fibrosis and grading of hepatic inflammation in children with chronic liver disease. In patients aged less than 3 years, it could differentiate not only significant fibrosis from no/minor fibrosis, but also significant inflammation from no/minor inflammation. According to the results of under the receiver operating curve for serum GP73 and APRI, the diagnostic value of serum GP73 for significant fibrosis is comparable to APRI.

Clinically, fibrosis is usually observed to be accompanied by inflammation. Iftikhar and colleagues first illustrated that the primary triggers of GP73 expression is fibrosis in chronic liver disease and necroinflammatory activity in acute liver diseases^18^. More and more studies confirmed that increased levels of serum GP73 was triggered in response to both inflammation and fibrosis^21,24^ and serum GP73 was positively associated with the progression of liver diseases^15,25^, which was also observed in our present studies. However, inflammation is found to be more closely correlated with serum GP73 levels, as significant differences were found in both age subgroups. These findings suggested that hepatic inflammatory activity may represent a primary driving force for the increase of serum GP73. Consequently, hepatic inflammatory grades could significantly influence the accuracy of serum GP73 in assessing hepatic fibrosis stages in children.

Based on the above results, the levels of serum GP73 was positively correlated with the progression of fibrosis and inflammation, both of which were signs for hepatic injury. Since increased ALT and AST levels are the most commonly used indicators of hepatocyte injury, while decreased serum ALB levels reflect impaired hepatic synthetic function^25^. These biomarkers are applicable both in child and adult. We thus investigated the relationship between serum GP73 and liver function indices. Our results revealed that serum GP73 levels were negatively correlated with ALB levels, but positively correlated with TB, ALT and AST levels, which was consistent with previous report in adults^19,25^. Furthermore, in patients with advanced fibrosis, serum GP73 was more significantly correlated with ALB and TB, suggesting that higher serum GP73 levels reflect advanced fibrosis. ALT and AST levels were not strongly correlated with advanced fibrosis when compared to patients with minor fibrosis. These maybe due to lower ALT and AST levels and higher TB levels in advanced fibrosis.

Distribution of clinical diagnosis is significantly different between age groups. Intrahepatic cholestasis (64.7%) was the most common diagnosis in patients aged less than 3 years, while hepatocyte injury (76.1%) was most common in patients aged over 3 years. Previous studies indicated that fibrogenesis was a common feature of hepatocyte response to different etiologies in chronic liver diseases^18^, and the up-regulation of serum GP73 was not affected by different diseases^20^. In addition, liver fibrosis caused by various etiologies usually had the same scoring system both in adult and children, except for nonalcoholic fatty liver disease (NAFLD)^26,27^. Therefore, different etiologies might not result in the alteration of serum GP73; however, further evaluation and more verification should be carried out in large multicenter-based cohorts.

The limitation of this study is that patients in significant fibrosis were younger than that in no/minor fibrosis, this could be further resolved by enlarging samples through multi-center cooperation.

In conclusion, serum GP73 level is significantly elevated in patients with chronic liver diseases, and closely correlated with liver pathological grading and staging. Nevertheless, serum GP73 may be significantly affected by age and inflammation, thus the application of it as a marker in fibrosis will be benefited through research in a larger samples and combination with other biomarkers.

## Material and Methods

### Study design

Between March 2014 and January 2017, 183 consecutive patients who received liver biopsy in our medical centers (Jinshan Hospital of Fudan University and Children’s Hospital of Fudan University) with various types of liver diseases were admitted. The indication for liver biopsy in patients was shown in Table 3. In patients aged less than 3 years, the most common clinical diagnosis was intrahepatic cholestasis, followed by hepatic dysfunction, glycogen storage disease, and hepatitis B. While the most common diagnosis in patients aged 3 years and above was hepatic dysfunction, followed by intrahepatic cholestasis, glycogen storage disease, hepatitis B, and Wilson’s disease. Eighty-six children who received hepatitis B screening between December 2009 and July 2015 were enrolled as healthy controls. All study participants were younger than 18. This study was approved by the Ethics Committee of Jinshan Hospital and Children’s Hospital and informed consent was obtained from the participants’ parents. All serum samples were collected and stored at −80 °C before this study. All methods relating to humans were performed in accordance with the relevant guidelines and regulations in our study.

**Table 3:** Clinical diagnosis when liver biopsy performed in patients aged less than 3 years and 3 years or older.

Clinical diagnosis	Age < 3 y	Age ≥ 3 y	P-Value
Intrahepatic cholestasis	75 (64.7%)	16 (23.9%)	<0.001
Hepatic dysfunction	25 (21.6%)	34 (50.8%)	<0.001
Glycogen storage disease	12 (10.3%)	7 (10.4%)	0.982
Wilson’s disease	0 (0%)	3 (4.5%)	0.046
Hepatitis B	4 (3.4%)	7 (10.4%)	0.102

Exclusion criteria were: (1) aged over 18 years old; (2) patients with renal diseases; (3) time between liver biopsy and serum collection exceed 2 weeks; (4) patients without liver function test at that time; (5) patients with biliary atresia.

### Measurement of serum GP73 level

Quantitative detection of serum GP73 was performed in Peking University Health Science Center, in unaware of the sample’s clinical and pathological information, by using commercially available double-antibody sandwich enzyme-linked immunosorbent assay (ELISA) kit (Hotgen Biotech Inc., Beijing, China), according to the manufacturer’s protocol.

### Liver histology

**Figure 3:**
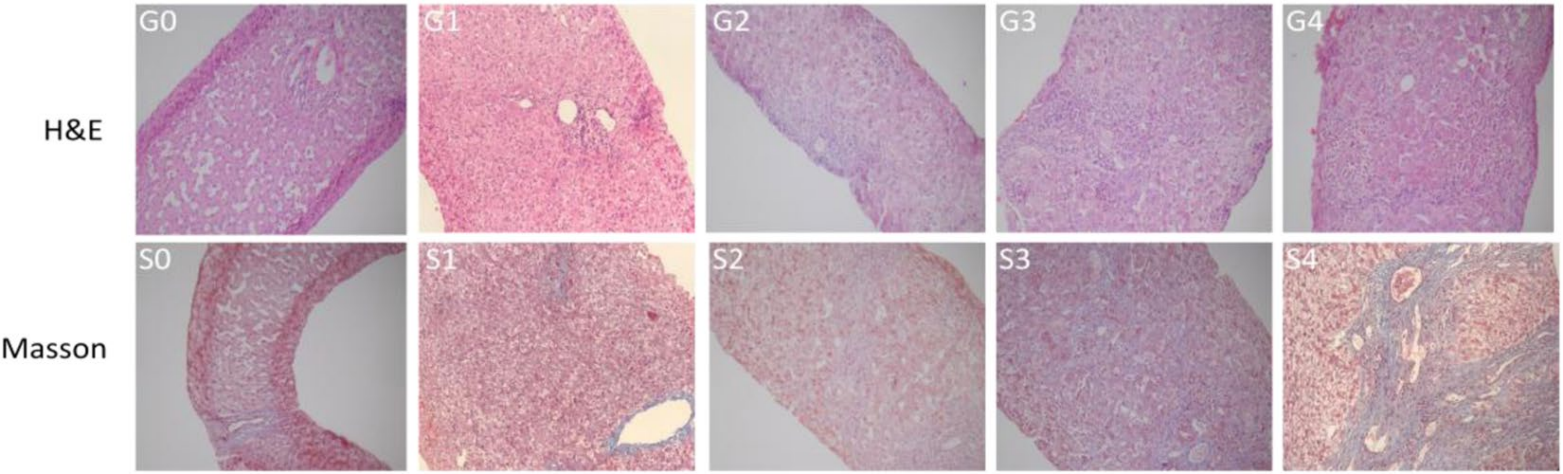
Hepatic inflammation activity grades and fibrosis stages in patients (original magnification, all images, x200). H&E staining in patients showed hepatic inflammation activity grades from G0 to G4. Masson’s staining in patients showed liver fibrosis stages from S0 to S4.

All liver biopsies were stained with hematoxylineosin, Masson’ trichrome, and reticular fiber stain. We adopted Scheuer’s scoring system which was modified in 2016^28^. Hepatic inflammation activity grade (G) was classified into G0 through G4; liver fibrosis stage (S) was classified into S0 through S4 (Fig. 3). No/minor inflammation was defined as an inflammation grade of “G0-G1”, significant inflammation was defined as an inflammation grade of “G2-G4”. No/minor fibrosis was defined as a fibrosis stage of “S0-S1”, significant fibrosis was defined as a fibrosis stage of “S2-S4”. Liver histopathology was evaluated by two independent pathologists in a double-blind manner. Another experienced pathologist was invited to judge and determine the final result if they had different results.

### Laboratory tests

Liver function tests, including total bilirubin (TB), alanine aminotransferase (ALT), aspartate transaminase (AST), serum albumin (ALB), r-glutamyl transpeptidase (GGT) and total bile acid (TBA), were measured using Beckman AU800 chemistry analyzer at two laboratories of Jinshan Hospital and Children’s Hospital, respectively. Whole blood routine such as platelet count was also measured at the above labs.

### APRI scores

Aspartate transaminase to platelet ratio index (APRI)^29^ was calculated based on the results of liver function tests and whole blood count during the admission for liver biopsy.

### Statistical analysis

Statistical analysis was performed using the IBM SPSS Statistics version 19 and GraphPad Prism 5.0. Measured data were expressed as mean ± standard deviation. Student’s t-test and ANOVA analysis were used to compare the difference between groups when the data satisfied the homogeneity of variance; otherwise, Mann-Whitney and Kruskal-Wallis tests were used. Partial correlation analysis was calculated. Rates of classification data were compared by χ^2^ square test. The diagnostic performance of serum GP73 was performed using area under the receiver operating characteristic (ROC) curve with 95% confidence interval (CI). P-values < 0.05 were considered to be statistically significant.
